# PostCOVID effect on endothelial function in hypertensive patients: A new research opportunity

**DOI:** 10.1111/jch.14376

**Published:** 2021-11-16

**Authors:** Luis Giménez‐Miranda, Luis Beltrán‐Romero, David León‐Jimenez, Pablo Stiefel

**Affiliations:** ^1^ Unidad Clínica de Atención Médica Integral (UCAMI) y Laboratorio de Epidemiología Clínica y Riesgo Vascular. Instituto de Biomedicina de Sevilla (IBiS) Hospital Universitario Virgen del Rocío/CSIC/Universidad de Sevilla Seville Spain

**Keywords:** circulating free DNA, endothelial dysfunction, endothelial microparticles, Laser‐Doppler flowmetry, SARS CoV‐2

## Abstract

SARS‐CoV‐2 is causing devastation both in human lives and economic resources. When the world seems to start overcoming the pandemics scourge, the threat of long‐term complications of COVID‐19 is rising. Reports show that some of these long‐term effects may contribute to the main cause of morbimortality worldwide: the vascular diseases. Given the evidence of damage in the endothelial cells due to SARS‐CoV‐2 and that endothelial dysfunction precedes the development of arteriosclerosis, the authors propose to measure endothelial function around 6–12 months after acute disease in hypertensive patients, especially if they have other cardiovascular risk factors or overt vascular disease. The methods the authors propose are cost‐effective and can be made available to any hypertension unit. These methods could be the “in vivo” assessment of endothelial function by flow mediated vasodilatation after ischemia by Laser‐Doppler flowmetry and the measurement of plasma free circulating DNA and microparticles of endothelial origin.

## 1 INTRODUCTION

A recent position paper of the European Society of Cardiology (ESC) Working Group for Atherosclerosis and Vascular Biology, and the ESC Council of Basic Cardiovascular Science states that (1) “Further research is urgently needed to combat the COVID‐19 pandemic and we emphasize that the role of vascular endothelium requires close scrutiny. There are today several outstanding questions that need to be addressed to elucidate more precisely the role of endothelial cells in COVID‐19 and to investigate potential routes to clinical translation” and (2) “A better understanding of the effects of SARS‐CoV‐2 on endothelial biology in both the micro‐ and macro‐vasculature is required, and endothelial function testing should be considered in the follow‐up of convalescent COVID‐19 patients for early detection of long‐ term cardiovascular complications”.^1^

In this respect, endothelial dysfunction (ED) is thought to be a primary step in atherosclerosis and cardiovascular disease. Several cardiovascular risk factors promote endothelial dysfunction including hypertension, obesity or insulin resistance among others. ED quantification has become a risk marker that predicts the development of cardiovascular disease and cardiovascular disease‐related mortality.^2^

## 2 MEASURENMENT OF ENDOTHELIAL FUNCTION

### 2.1 Flow mediated vasodilatation in response to the ischemia

Endothelial function can be measured by assessing the hyperemic response to ischemia caused by inflating a blood pressure (BP) cuff, 20 mm Hg above the patient's systolic BP. The magnitude of the response can be assessed either by high‐resolution ultrasonography or plethysmography.^3^

One of the most recent and innovative techniques to assess the magnitude of response is Laser‐Doppler flowmetry, which mainly allows the determination of the microcirculation status. This is a noninvasive technique, but its measurements are probably more independent of the observer since the results are automatically obtained by software. This software assesses many parameters both in general and adjusted analysis of the response. Although some parameters measure the speed of the response, such as the slope or the time to maximum hyperemia, others are related to the duration of this response, such as the time to reach the half value after the maximum hyperemia. A previous study of our group found that the area of hyperemia was the parameter with higher sensitivity and specificity for identification of patients with coronary artery disease, because the area depends on the speed as well as on the intensity and duration of the response.^4^

### 2.2 Cell‐free DNA measurement

Although initially related to neoplastic diseases cell‐free DNA (c‐fDNA) levels, has more recently been related to pathologies involving ischemia such as acute coronary syndrome,^5^ ischemic heart failure,^6^ stroke,^7^ and mesenteric ischemia,^8^ as well as in patients who have suffered cardiac arrest outside the hospital.^9^ Similarly, increases in circulating c‐fDNA have been documented in situations involving hypoxia, such as experimental acute pulmonary thromboembolism^10^ or obstructive sleep apnea/ hypopnea syndrome.^11^

Our group have also observed higher levels of c‐fDNA in patients with preeclampsia (PCL, a disease related to placental ischemia) that also increases with the severity of disease, being higher in patients with HELLP syndrome, acronym Hemolysis, Elevated Liver enzymes and Low Platelet count. According to our data, we proposed a cutoff point of 950 ng/ml of c‐fDNA as being suspicious of severe illness because these values had high sensitivity and specificity for detecting severe PCL and HELLP syndrome^12^, ^13^ (Figure 2).

Finally, Alvarado Vasquez and coworkers^14^ have described that circulating cell‐free mitochondrial DNA might be the probable inducer of early endothelial dysfunction.

### 2.3 Circulating endothelial microparticles

Circulating microparticles (MPs) are small vesicles that are released in response to several injuries. The level of circulating MPs in peripheral blood has been reported to be increased in cerebrovascular disease, hypertension, diabetes, smoking, coronary disease, and obstructive sleep apnea (OSA) syndrome. There exists a positive correlation between circulating levels of MPs and nocturnal hypoxemia severity.^15^ We have also previously reported that changes in MPs after continuous positive airway pressure in OSA patients were greater in those with a more severe disease, defined according to the oxygen desaturation and apnea‐hypopnea indexes, suggesting that in more severe patients the benefit is greater.^16^ Finally, Sinning and coworkers^17^ determined CD31+/Annexin V+ MPs by flow cytometry in 200 patients (age 66.1 + 10.4 years) with angiographically proven stable coronary artery disease and correlated with cardiovascular outcomes. The median follow‐up time for major adverse cardiovascular and cerebral events (MACCE) was 6.1 (6.0/6.4) years. A first MACCE occurred in 72 patients (37%). MPs levels were significantly higher in patients with MACCE compared with patients without event (*p* =0.004). In multivariate analysis (cardiovascular risk factors, number of diseased vessels, use of angiotensin‐converting enzyme‐inhibitors, and statins), high MPs level were associated with a higher risk for cardiovascular death [Hazard ratio (HR) 4.0, 95% confidence interval (CI) 1.1–14.6; *p* = .04], the need for revascularization (HR 2.4, 95% CI 1.3–4.4; *p* = .005), and the occurrence of a first MACCE (HR 2.3, 95% CI 1.4–3.8; *p* < .001). Inclusion of the MP level into a classical risk factor model substantially increased c‐statistics from 0.637 (95% CI: 0.557–0.717) to 0.702 (95% CI: 0.625–0.780) (*p* = .03). Therefore, they concluded that the level of circulating CD31+/Annexin V+ MPS is an independent predictor of cardiovascular events in stable coronary patients and may be useful for risk stratification.

## 3 CLINICAL AVAILABILITY OF THE AFOREMENTIONED ENDOTHELIAL FUNCTION ASSESSMENT

A Laser‐Doppler flowmeter is a relatively inexpensive device (around 20,000 euros) easy to handle by any nurse after minimal training. It is also relatively well tolerated by the patient and could be done in about 20 min including the time necessary for relaxing the patient. It only requires a comfortable chair in a low‐stress environment and, therefore, it could be used in any hypertension unit, where, on the other hand, we attend patients who, apart from hypertension, usually have other factors of vascular risk and even overt vascular diseases (secondary prevention)

Measurement of cfDNA is used in the clinical routine for early cancer detection, detection of recurrence in localized cancer, prediction of response to treatment in metastatic cancer, identification of resistance mechanisms in refractory cancers, and monitoring the response in metastatic cancer. In addition, it is used to monitor the treatment of systemic lupus erythematosus. It is also a biomarker for solid organ transplant rejection. Moreover, it is also used to evaluate the prognosis and monitoring of traumatisms.

Circulating free fetal DNA is routinely analyzed to determine the sex of the fetus, the fetal RhD factor when the mother is Rh‐ to rule out hemolytic disease of the newborn, for diagnosis of fetal monogenic diseases, dominant inheritance diseases, recessive inheritance diseases, and fetal aneuploidy studies.

The measurement of circulating microparticles requires only trained personnel and a flow cytometer that is available in most hospitals for the diagnosis or follow‐up of pathologies such as leukemia, lymphoma, primary immunodeficiency, monitoring of the hematological status of patients with HIV infection, as well as the detection of any cell line.

As these research studies will be on human beings, local ethics committee approval must be obtained and patients should be informed of the possible adverse effects of these techniques which will include those derived from venipuncture and keeping the cuff inflated on the arm for 4 min.

## 4 THE NEW PROPOSAL

**FIGURE 1:**
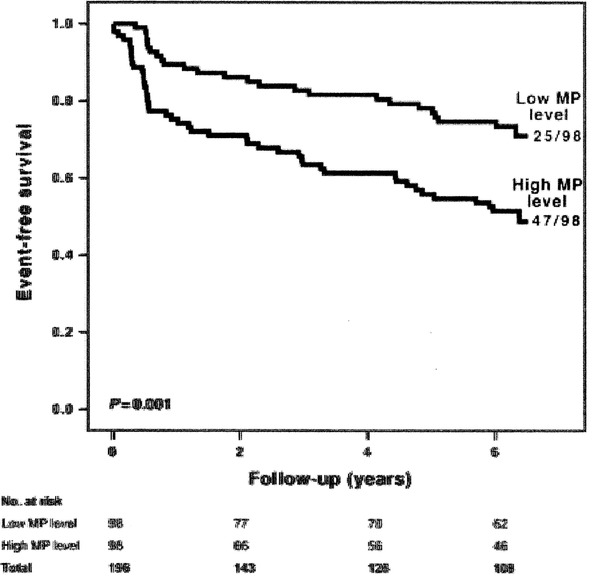
Endothelial microparticles in stable coronary artery disease and time for major adverse cardiovascular and cerebral events (taken from Eur Heart J 2011; 32(16):2034‐41 with permission of the author)

Practically all hypertension units are located in hospitals that have or could have the three techniques mentioned above. The finding by Sinning and coworkers that is reproduced in Figure 1 (with the author's permission) shows how, from practically the first 6–12 months, both curves (those that present events and those that do not) begin to differ.

**FIGURE 2:**
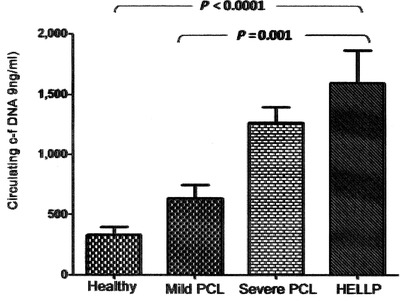
Circulating c‐fDNA in controls and patients with preeclampsia according to the severity of disease (taken from Am J Hypertens 2013; 26 (12): 1377–80)

Our proposal is very simple: all hypertensive patients who have suffered an episode of SARS‐CoV‐2 infection ‐and even more so if they have other associated vascular risk factors or already manifested vascular disease‐ should be evaluated from the point of view of their endothelial function at least once 6–12 months after the episode, by any of the three aforementioned methods.

We encourage all interested researchers.to design a comparative study, age‐sex‐risk factor matched study of hypertensive patients with and without history of SARS‐CoV‐2. Both groups should be compared to decide whether there are significant differences, and in such case, establish cut‐off points from which to establish measures aimed at improving the future vascular prognosis in infected patients who have worsened their vascular risk due to deterioration of endothelial function.

## CONFLICTS OF INTEREST

There is not any conflict of interest.

## FUNDING STATEMENT

This work was supported by Consejeria de Salud, Junta de Andalucía, Spain [grant number PI 0456‐2018].
